# The role of digital and AI-based tools in music theory education

**DOI:** 10.1371/journal.pone.0352494

**Published:** 2026-11-06

**Authors:** Ali Kalkan

**Affiliations:** Ankara Music and Fine Arts University, Ankara, Türkiye; Mae Fah Luang University School of Anti Aging and Regenerative Medicine, THAILAND

## Abstract

This study examined the role of digital platforms and artificial intelligence in music theory instruction from the perspectives of both students and instructors. Using a convergent mixed-methods design, data were collected through questionnaires administered to 47 undergraduate students and 9 instructors. Quantitative responses were analyzed descriptively, while open-ended responses were examined through inductive thematic analysis. The findings indicated that students associated these tools with increased engagement, perceived support for understanding complex theoretical concepts, and individualized learning opportunities. Written responses further suggested that flexible access and visual representations were particularly valuable in facilitating comprehension. Instructors, however, emphasized the importance of clear content, accurate information, and an awareness of practical limitations in classroom implementation. Although both groups acknowledged the potential of these technologies, their reasons for valuing them differed. Overall, the findings suggest that digital and AI-based tools may be particularly valuable when they are meaningfully integrated into everyday teaching practice, rather than being used as stand-alone additions. Their perceived educational value appears to depend not only on technical sophistication but also on alignment with instructional goals. Teacher readiness also appeared to be an important consideration in the effective integration of these tools. As automation assumes an increasingly prominent role in education, adapting resources to diverse learning needs appears to be of growing importance.

## Introduction

Students follow different learning pathways when engaging with musical material, whether through aural activities or instrumental practice; musical practice strategies have been reported to vary considerably according to individual needs [1]. This is a natural process, as in other areas of learning, and individuals may differ in how they understand and process information. However, recognizing, interpreting, or explaining on the musical score the notes that students hear or play can be one of the challenging aspects of learning music theory. In this context, approaches to musical literacy have emphasized the importance of moving beyond a narrow focus on sight-reading and dictation in aural skills instruction by incorporating contextual listening and creative music-making and by developing aural understanding before visual representation [2]. Similarly, aural preparation based on hearing, performing, analyzing, and creating rhythms and pitches before introducing notation may play a supportive role in the development of music literacy skills [3]. Particularly in the early stages of music learning, students may use less systematic practice strategies, and repetitive practice does not always result in effective learning [4]. Students may also differ in their performance on aural skills, and these differences may be associated with various perceptual and cognitive characteristics [5]. Although students may be able to perceive certain musical structures aurally, they may have difficulty explaining what they know using music theory terminology [6]. Nevertheless, repetition is a commonly used strategy when students work with musical material, while effective music practice involves self-regulatory processes such as planning, identifying problems, developing strategies, and evaluating progress [7].

Contemporary learning environments are changing as digital technologies provide new possibilities for teaching and learning in music education [8]. One example of this transformation in music theory instruction is the flipped classroom approach, which can support pre-class preparation, in-class activities, and post-class learning through digital learning environments [9]. Computer-assisted learning systems can allow students to progress at their own pace and enable the learning process to be adapted to individual rates of progress [10]. Findings suggest that these resources can go beyond simply providing access to content. As digital tools become part of students’ practice routines, they can support music practice, motivation, and self-regulated learning processes, although their effects may vary across students [11]. The integration of technology into instructional settings, therefore, involves more than simply adding new tools; software used in music education can provide various opportunities to support teaching and learning processes [12]. There is also evidence that technology-supported instruction can support music learning. For example, students who used technology-supported materials to learn musical notation were reported to have higher academic achievement than students who received traditional instruction [13]. Digital platforms for music theory and aural skills can also provide a complementary channel for learning, particularly in online environments, while allowing student participation and study habits to be monitored [14].

In the context of the present study, digital tools refer broadly to software, online platforms, and technology-based applications used to support music theory learning and teaching, including notation software, aural-training applications, interactive music-learning platforms, and related online resources (e.g., MuseScore, Finale, EarMaster, and Chrome Music Lab). AI-based tools refer more specifically to applications that employ artificial intelligence to support functions such as assessment and evaluation, adaptive and personalized learning, and intelligent tutoring, which represent established areas of AI application in education [15]. Within this broader technological context, generative AI (GenAI) refers to AI technology that automatically generates new content in response to user prompts, including text, images, music, and software code [16]. In the present study, ChatGPT is considered within this generative AI category.

Artificial Intelligence (AI) in education can support teaching and learning through a range of applications, including adaptive and personalized learning, assessment, and intelligent tutoring systems [15]. AI-based systems can adapt learning content to individual needs based on learner models and provide feedback to students [15]. In music education, AI-supported personalized instruction can provide materials and feedback according to students’ individual learning needs, and such applications have been associated with positive outcomes in musical skills, emotional expression, and student satisfaction [17]. Research on mobile learning similarly suggests that instruction supported by mobile technologies can contribute to achievement in basic music theory [18]. At the same time, recent research indicates that the educational value of AI-based tools should be considered not only in terms of their technological capacities but also in relation to how they are pedagogically and didactically integrated into teaching and learning processes [19].

The availability of digital tools alone does not guarantee their effective use in educational settings [20]. Instructors’ attitudes toward and acceptance of technology can shape how they adopt and use these tools in their teaching practices [21]. In music theory education, digital resources can provide opportunities for students to revisit theoretical concepts and reinforce related knowledge and skills through practice [18]. The ways in which instructors integrate these tools into their courses may also play a role in shaping students’ experiences and perceptions of AI-supported learning activities [22]. In online music education, it is therefore important to structure technological opportunities pedagogically while maintaining the instructor’s guiding role in the learning process [23].

Digital technologies are becoming an increasingly common part of music education environments as technology advances and access expands [20]. Their use in music education also raises challenges related to teachers’ digital competence, technological access, student participation, and inclusion [24]. Digital technologies can also shape how university students engage in creative music-making and collaborative composition processes [25]. However, the literature on AI-supported music teaching in higher education remains fragmented, with studies directly related to music theory representing a more limited part of this literature [26]. In particular, research that considers both student and instructor perspectives on the perceptions and use of digital and AI-supported tools in university-level music theory education remains underrepresented.

To address this gap, the present study examines how digital systems and AI are used in university-level music theory education from both student and instructor perspectives. Because such practices may vary across different learning contexts, how participants interpret these technologies is particularly important. Accordingly, the study focuses on perceptions of digital tools, reported patterns of their use in the classroom, and how students perceive their potential influence on their engagement with music theory concepts.

The study was guided by the following research questions:

1. How do students perceive digital and AI-based tools, and what learning experiences do they report when using these tools in music theory education?
2. For what purposes and within what limitations do instructors use these technologies in music theory teaching?
3. How do students and instructors perceive the pedagogical effects of these tools on learning processes, conceptual understanding, and motivation?
4. What similarities and differences emerge between student and instructor perspectives?

By examining these questions, the study focuses not only on the digital tools themselves but also on the pedagogical assumptions that shape how technology is integrated into the teaching and learning of music theory, thereby contributing to ongoing discussions in the field of music technology education.

### Pedagogical foundations of music theory education

When learning music theory, it may not be sufficient for students to know only notation, chords, rhythm, counterpoint, or other theoretical concepts. Relating this knowledge to musical structures that are performed and applied can also be considered an important dimension of learning music theory [27]. Relating theoretically learned musical structures to listening and practical musical activities is important for enabling students to use this knowledge in different musical contexts [2]. Therefore, how the relationship between theoretical knowledge and aural and practical studies is established is important in music theory education.

In music theory courses, activities based on analysis, listening, and performance may enable students to use the theoretical knowledge they have learned in different musical situations and structures. Rather than theoretical structures remaining only at the level of definitions, hearing, analyzing, and applying them may support the establishment of connections between theoretical knowledge and musical practice [28]. However, practice and repetition alone cannot be said to result in effective learning. Identifying the problem encountered, planning a practice strategy accordingly, and evaluating one’s own practice are also part of the self-regulation process in musical practice [7]. This process may not be the same for every student or individual. Students may differ in their musical experiences, individual abilities, and particularly the practice strategies they use [1]. The amount of practice time and the strategies students use when reinforcing a concept or performance may also vary; different strategies such as repetition, analytical practice, listening, and error correction may form part of the musical practice process [4]. Therefore, it is important to provide learning opportunities in music theory education that can accommodate students’ different learning situations. Digital learning environments may also enable students to revisit course materials at different times and continue their individual study; flipped classroom practices in music theory courses have been reported to facilitate students’ access to preparatory videos and materials [29].

### Educational use of digital and ai-based technologies in music education

Digital technologies can be used in various ways in music education for listening, practice, music production, and individual study processes [8]. Digital tools may support students in setting goals and monitoring their progress during individual music practice; they may also facilitate receiving guidance from their teachers between lessons [30]. These opportunities indicate that digital technologies can be used not only to access musical content but also to support students’ musical practice and learning processes.

The use of digital technologies in music education may enable students not only to access musical content but also to participate in music production and collaborative processes [25]. Therefore, the use of digital tools in educational settings should not be regarded merely as adding technology to existing teaching practices. The role of these tools in the learning process should be considered in relation to how students participate in musical activities and how technology is integrated into the teaching process [31].

AI-supported technologies offer various opportunities to support students’ individual progress and learning processes in music and music theory education. AI-based systems and digital platforms can provide content and feedback adapted to learning processes [20,32]. However, these systems and structures may be regarded not as tools that replace instructors, but as technologies that support the teaching and learning process. Therefore, the use of AI- and digital platform-supported tools in music and music theory education should be considered not only in terms of their technological features but also in terms of how they are related to students’ learning needs and pedagogical processes [19].

The use of digital tools in music education may also be related to instructors’ attitudes toward, interest in, and competence with these technologies. Instructors’ perceptions of technology as useful and usable may influence how and for how long these tools are used in teaching and learning processes. A study conducted with music teachers found that technology acceptance directly influenced technology use, while technological competence had an indirect effect through technology acceptance [21].

### Research gap

The use of digital and AI-supported technologies in music education has been addressed in recent years within different teaching and learning processes [33]. These tools can be used for various purposes, including individual study, feedback, music production, collaboration, and access to learning materials [25,33]. However, the mere presence of technology in the educational process does not necessarily imply a pedagogical contribution [34]. How instructors integrate technology into their courses and how the tools used are related to students’ learning needs are also important [35].

Research on digital technologies and artificial intelligence in music education has been increasing, and various applications for music theory teaching are also represented within this body of research [20,36]. However, existing research appears to be predominantly student-centered, with the teacher dimension receiving more limited attention [36]. Zhang [36] reported that 40.7% of the studies reviewed focused on students, whereas studies examining students and teachers together accounted for 14.8%. Therefore, examining the perspectives of students and instructors together within the same learning environment may contribute to understanding the similarities and differences between the two groups’ evaluations of the use of digital and AI-supported technologies.

Based on this need, the present study examines the use of digital and AI-supported tools in university-level music theory education from the perspectives of students and instructors. The study focuses not only on which tools are used, but also on students’ learning experiences with these tools, instructors’ purposes for using them, and both groups’ evaluations of their pedagogical contributions and limitations. Thus, the study aims to evaluate the role of digital and AI-supported tools in music theory education not only in terms of their technological features, but also through the experiences and perceptions of the students and instructors who use these tools.

## Methodology

This study was conducted within the framework of a convergent mixed-methods design to examine student and instructor perspectives on the use of digital and AI-supported tools in university-level music theory education [37]. Quantitative and qualitative data were collected in parallel during the same data collection period, and equal importance was given to both types of data in the study. While the quantitative data enabled a descriptive examination of participants’ views and usage tendencies regarding digital and AI-supported tools, the qualitative data obtained from open-ended questions allowed for a more detailed examination of participants’ experiences and evaluations. In a convergent mixed-methods design, analyzing the quantitative and qualitative components separately and subsequently bringing the findings together for evaluation allows different types of data to be related within the framework of the same research problem [38].

Quantitative and qualitative data were obtained simultaneously through closed- and open-ended questions included in questionnaires administered to students and instructors via Google Forms. While the data obtained from the closed-ended questions were analyzed descriptively, the open-ended responses were coded according to similarities in their content and examined under categories. Thus, the aim was to evaluate the trends emerging from participants’ responses to the closed-ended questions together with the views and experiences expressed in the open-ended questions.

### Research design

The research focused on descriptively examining student and instructor views regarding the use of digital and AI-based tools in university-level music theory education and on examining the experiences of these two participant groups in practice. In the quantitative component, participants’ views regarding the use of these tools and their usage tendencies were examined through the frequency distributions of responses to closed-ended questionnaire items. In the qualitative component, participants’ responses to open-ended questions were used to examine in detail their experiences with digital and AI-supported tools, their perceived benefits and limitations, and their perceived advantages and disadvantages.

The quantitative and qualitative components were initially analyzed separately, after which the relevant findings were evaluated together within the framework of answering the research questions. The categories derived from the open-ended responses of students and instructors were also compared across the groups to identify and examine similarities and differences between the perspectives of the two groups. This approach is consistent with the mixed-methods principle of bringing together different types of data after analysis for comparison and integrated interpretation [38].

### Participants

Undergraduate students enrolled in music theory courses and instructors teaching in this field participated in the study. Questionnaire links prepared via Google Forms were distributed to the participants, and participation in the study was voluntary. Data collection was conducted between October 1, 2024, and January 25, 2025. A total of 47 students and 9 instructors participated in the study. The distributions of the participants’ demographic characteristics are presented in Table 1.

**Table 1:** Demographic information.

Group	Variable	Category	Frequency (n)	Percentage (%)
Students	Gender	Female	28	59.6
		Male	19	40.4
	Age Group	18–19 years	22	46.8
		20 years	10	21.3
		21 years	7	14.9
		22 years	4	8.5
		23–24 years	4	8.5
Instructors	Gender	Female	3	33.3
		Male	6	66.7
	Years of Experience	5–9 years	2	22.2
		10–14 years	3	33.3
		15–19 years	2	22.2
		20 years and above	2	22.2

According to Table 1, the student group consisted of 28 female (59.6%) and 19 male (40.4%) participants. The students ranged in age from 18 to 24 years. The instructor group consisted of three female (33.3%) and six male (66.7%) participants. The instructors’ professional experience ranged from 5–9 years to 20 years or more.

### Ethics statement

This study was approved by the Ethics Committee of Ankara Music and Fine Arts University (Approval No. 2024/65; Decision Date: 23 October 2024). Participation was voluntary. Before accessing the questionnaire, all participants were provided with information about the study and were required to indicate their decision to participate through an electronic consent item. Written informed consent was therefore obtained electronically from all participants prior to data collection.

### Data collection instruments

Two separate questionnaires developed by the researcher were used for students and instructors in the study. The questionnaire items were developed in accordance with the aims and research questions of the study to identify participants’ use of digital and AI-supported tools, their experiences, perceived benefits and limitations, and their views regarding the use of these tools in music theory education. Depending on the nature of the research questions, the questionnaires included demographic questions, categorical response options, frequency-of-use and agreement-based rating items, and open-ended questions. During the questionnaire development process, the alignment of the items with the purpose of the study, their clarity, and their comprehensibility for the target participant groups were taken into consideration. In educational research, it is recommended that questionnaire items be formulated clearly and in accordance with the intended purpose, subjected to expert review, and reviewed prior to administration [39].

To clarify the scope of digital and AI-supported tools without limiting participants to specific applications, the questionnaires included illustrative examples commonly used in music education (e.g., Chrome Music Lab, MuseScore, Finale, EarMaster, and ChatGPT).

### Student questionnaire

The student questionnaire included demographic questions, rating items, and open-ended questions. The demographic section included questions regarding age, gender, years of music theory education, and frequency of using digital and/or AI-supported tools. The rating items examined students’ views regarding their experiences with digital and AI-supported tools in music theory learning, including learning at their own pace, visualization of musical concepts, perceived conceptual understanding, motivation, remembering and making sense of learned topics, as well as perceived difficulties such as technical or mechanical use, limited Turkish-language content, distraction, and superficial engagement. The open-ended questions focused on the perceived contributions, difficulties and limitations, and ways of using digital and AI-supported tools in music theory learning.

### Instructor questionnaire

The instructor questionnaire consisted of questions designed to identify participants’ views and experiences regarding the use of digital and AI-supported tools in music theory teaching, in addition to their demographic and professional characteristics. The questionnaire addressed instructors’ use of these tools to support students’ individual study and practice, diversify harmonic analysis examples, and visually represent and explain musical structures. It also examined instructors’ views regarding technical infrastructure and pedagogical material limitations, as well as the possibility that students’ use of digital and AI-supported tools to obtain ready-made answers may lead to superficial learning. In addition, open-ended questions addressed the perceived contributions, difficulties and limitations of these tools, as well as instructors’ observations regarding how and for what purposes students use them in music theory learning. These questions allowed instructors to explain their views and experiences in greater detail in their own words. The full instructor questionnaire is provided as Supplementary Material in S3 File.

### Expert review

The initial drafts of the questionnaires were submitted for expert review to three academics who teach music theory courses. The experts were asked to evaluate the items in terms of their alignment with the purpose of the study, clarity of wording, and comprehensibility for the target participant groups. Based on the expert evaluations, items identified as requiring modification were reviewed, and the necessary revisions were made before the questionnaires were finalized. The expert review was used to examine the questionnaires in terms of their scope and content; this process was not considered a psychometric validation of the construct validity of the questionnaires.

### Pilot study

Prior to the final administration of the questionnaires, a pilot study was conducted with 10 students and two instructors who were not included in the main study sample. The pilot study was conducted to review the questionnaires before their final administration.

Although Cronbach’s alpha coefficients had been calculated for the student and instructor questionnaires in the previous analyses, these coefficients were not considered strong evidence of the psychometric reliability of the questionnaires because of the small pilot samples, particularly the instructor pilot group, which consisted of only two participants.

### Analysis

#### Quantitative analysis.

R and Python programming environments were used for the organization and analysis of the quantitative data. In the Python environment, the *pandas* library was used for data organization and processing, while *seaborn* was used for the visualization of the findings. *Seaborn* is a library developed for statistical data visualization in Python and can be used in integration with *pandas* data structures [40]. Frequency and percentage calculations were performed using R and Python.

Quantitative data obtained from the closed-ended questionnaire questions were analyzed descriptively and separately for the student and instructor groups. Frequency (*n*) and percentage (%) distributions were used to describe participants’ views, experiences, and usage tendencies regarding digital and AI-supported tools. Since the questionnaires included categorical, rating-based, and other response formats, the analyses were conducted in accordance with the response format of each question.

Because the student and instructor questionnaires had different question structures and the primary aim of the study was to describe participants’ views and experiences, responses from the two groups were not compared using a common scale score. Therefore, independent-samples *t*-tests, correlation analyses, or other inferential statistical tests were not performed. The quantitative findings represent participants’ self-reported perceptions and experiences and were not interpreted as objectively measured learning outcomes or directly observed classroom behaviors.

#### Qualitative analysis.

Qualitative data obtained from the open-ended questions were examined using an inductive thematic analysis approach to identify recurring patterns of meaning in participants’ experiences with digital and AI-supported tools, their perceived benefits, and the limitations they encountered. Thematic analysis is a flexible method that enables the identification, analysis, and reporting of patterns of meaning within qualitative data [41]. In an inductive approach, coding is driven by the data rather than being based on a predetermined coding framework [41].

During the analysis process, the open-ended responses of students and instructors were initially examined separately. After familiarization with the content of the responses, statements with similar meanings and content were coded; related codes were grouped under higher-order categories, and the extent to which these categories represented the responses in the dataset was reassessed. This process drew on Braun and Clarke’s [41] thematic analysis approach, which involves familiarization with the data, generation of initial codes, identification of patterns, and their subsequent review.

In addition to the researcher, three academics who teach music theory courses and who had also been consulted during the questionnaire development stage participated in the coding process. These academics evaluated the open-ended responses independently of one another. Following the independent evaluations, the coding was compared; codes with similar content were brought together to form categories, and the extent to which the categories represented participants’ responses was evaluated. Where differences occurred among the coders’ evaluations, the relevant responses and coding decisions were re-examined, and the final categories were determined by reaching a common evaluation.

The categories derived from the student and instructor responses were subsequently compared across groups to examine similarities and differences between the perspectives of the two participant groups. In thematic analysis, explicitly acknowledging the researcher’s active role in identifying and interpreting patterns is important for methodological transparency [41]. Accordingly, the categories identified through the analysis were treated not as predetermined structures but as analytical categories developed through the systematic examination of participants’ open-ended responses. The final categories and supporting participant statements are presented in the Findings section.

### Use of generative artificial intelligence (AI)

The manuscript was originally written entirely in Turkish by the author. During the translation of the manuscript into English, the author used ChatGPT (OpenAI) as a supporting tool to assist with translation, grammatical accuracy, clarity, and field-specific terminology. ChatGPT was also used to assist in writing R code for the graphical presentation of the findings. All AI-assisted translations, text, and code were reviewed and verified by the author. The research data, analytical decisions, interpretations, and conclusions remain the sole responsibility of the author.

### Findings

This section presents the quantitative and qualitative findings in relation to the four research questions. Quantitative findings are reported using frequencies and percentages, while qualitative findings are presented through the categories derived from the open-ended responses and representative participant statements.

### Student perceptions of digital and AI-based tools

Descriptive analysis of the student questionnaire indicated generally positive perceptions of digital and AI-supported tools in music theory education. Fig 1 presents the distribution of student responses for the selected questionnaire items related to these perceptions.

**Fig 1:**
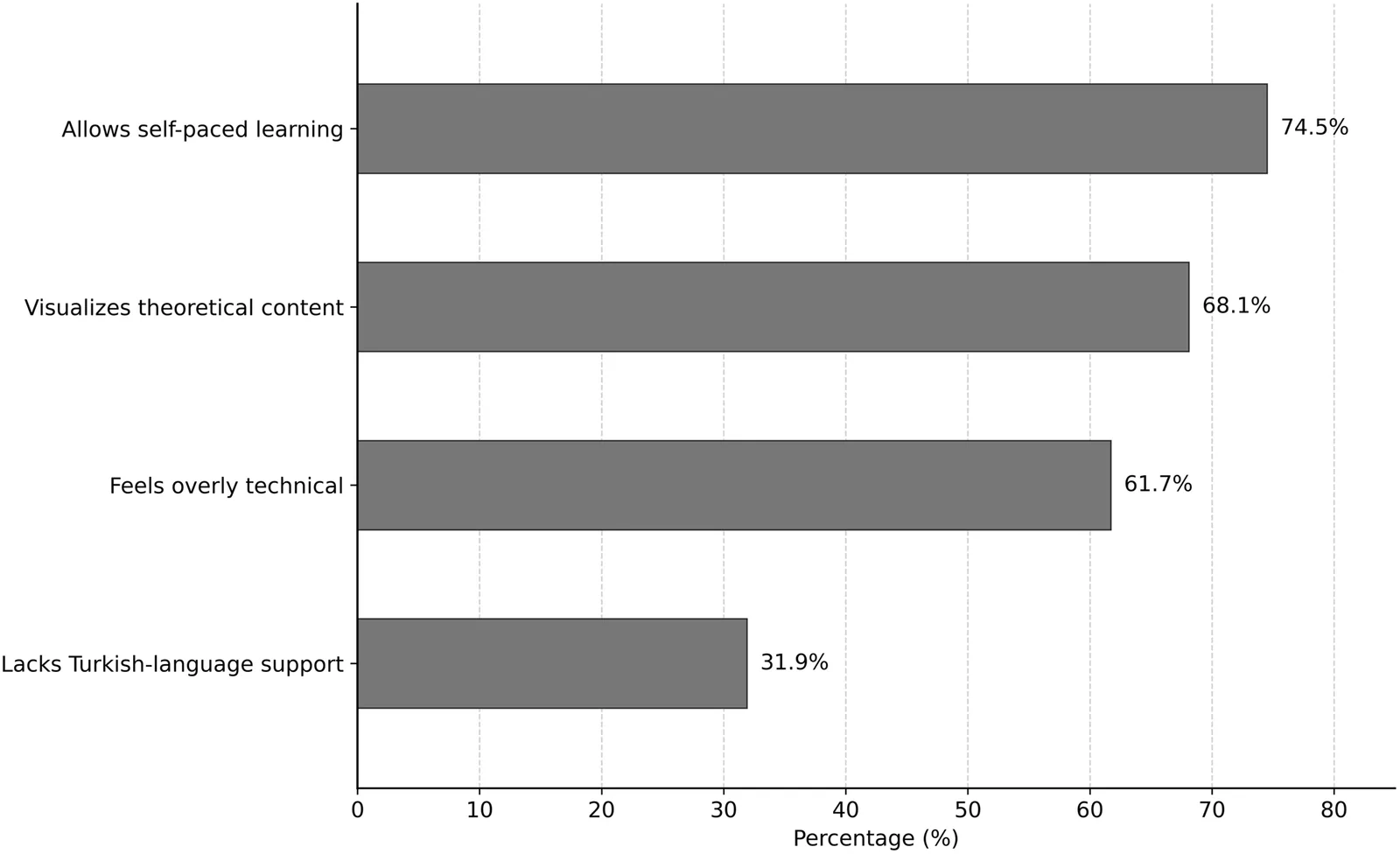
Student perceptions of digital and AI-based tools in music theory education.

Alt text for Fig 1: Bar chart showing student perceptions of digital and AI-based tools, including self-paced learning, visualization of theoretical content, technical complexity, and limited Turkish-language support.

For the questionnaire items presented in Fig 1, percentages were calculated according to the response categories of the respective items. The selected items represent student responses concerning perceived benefits and limitations of digital and AI-supported tools 74.5% of students reported that digital tools allow them to progress at their own learning pace.

- 68.1% indicated that digital platforms helped them visualize theoretical content.
- 61.7% agreed that some tools feel overly technical or mechanical.
- 31.9% reported difficulty due to limited Turkish-language content.

Inductive thematic analysis of the students’ open-ended responses identified three recurring categories:

1. Self-paced reinforcement
2. Conceptual visualization
3. Platform-related limitations

Example excerpts:

“When I worked on analyzing harmony sequences, I understood it faster than what was explained on the board.” (Student 14)

“Being able to repeat the same topic in different ways on the AI-supported platform makes me feel comfortable.” (Student 9)

These excerpts illustrate how students describe pacing and repetition features in relation to conceptual understanding.

### Teachers’ purposes of use and perceived limitations

Descriptive analysis of the instructor questionnaire indicated several self-reported purposes for using digital and AI-based tools in music theory teaching, as well as perceived limitations associated with their use. Fig 2 presents the distribution of instructor responses for the selected questionnaire items related to these purposes and limitations.

**Fig 2:**
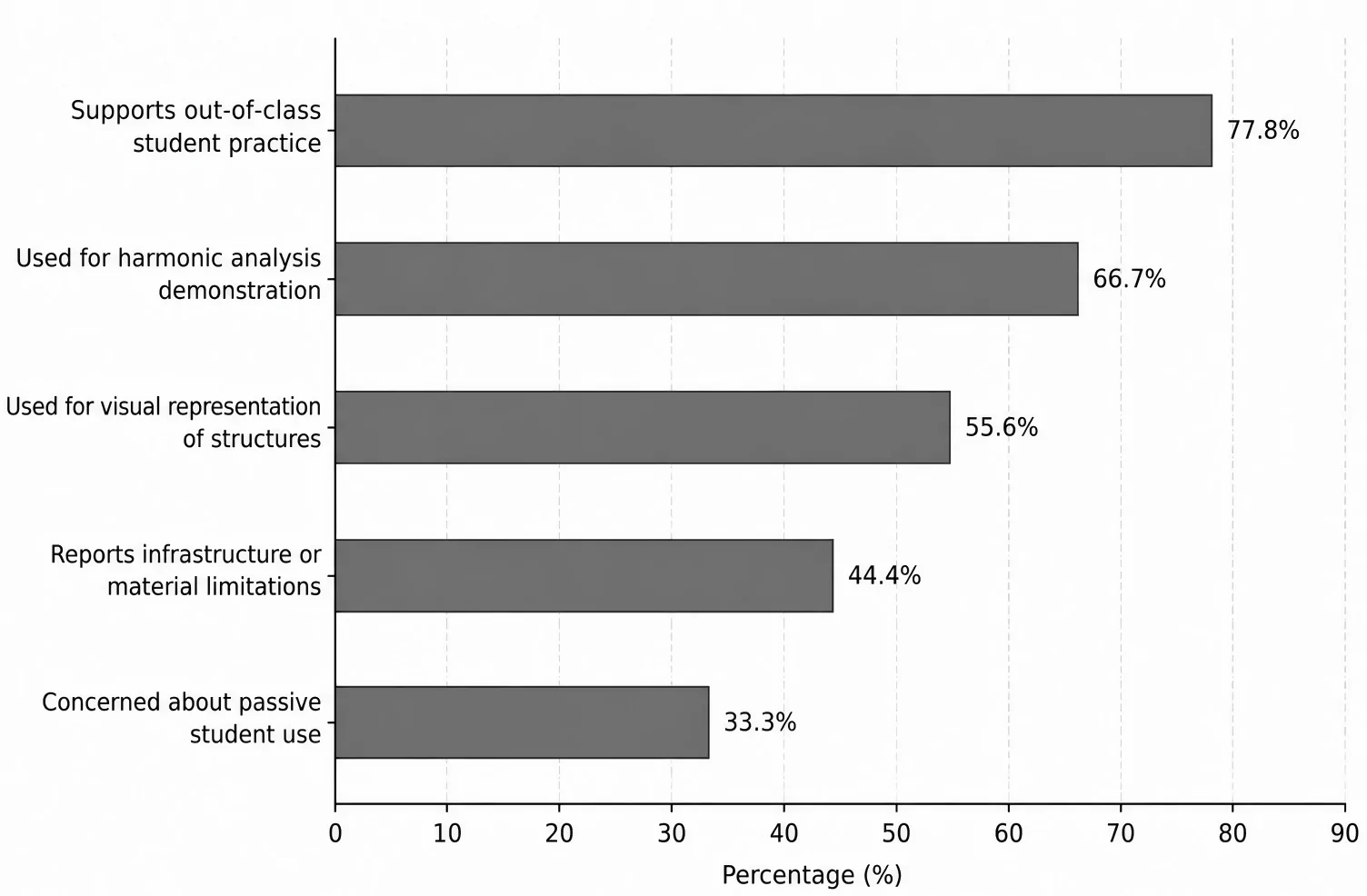
Instructor feedback on the use of digital and AI-based tools in music theory education.

Alt text for Fig 2: Bar chart showing instructor use of digital and AI-based tools, including support for practice, analysis, visualization, and reported limitations.

For the questionnaire items presented in Fig 2, the instructor responses were distributed as follows:

- **77.8%** reported using these tools to support students’ out-of-class practice.
- **66.7%** reported using them to diversify harmonic analysis examples.
- **55.6%** reported using them for visual representation of musical structures.
- **44.4%** identified infrastructure limitations or insufficient pedagogical materials.
- **33.3%** expressed concern that students may rely on tools for ready-made answers, potentially encouraging superficial learning.

Representative instructor responses include: “If there are technological tools but no content, those tools can be a waste of time.” (Teacher 5)

“Some students only use them to achieve results, and their intention to learn the process remains weak.” (Teacher 3)

### Student-reported perceptions of learning, motivation, and conceptual understanding

Student responses concerning perceived conceptual understanding, motivation, retention, and learning-related challenges were analyzed descriptively. Fig 3 presents the distribution of responses for the selected questionnaire items. These findings represent students’ self-reported perceptions and should not be interpreted as objectively measured learning outcomes.

**Fig 3:**
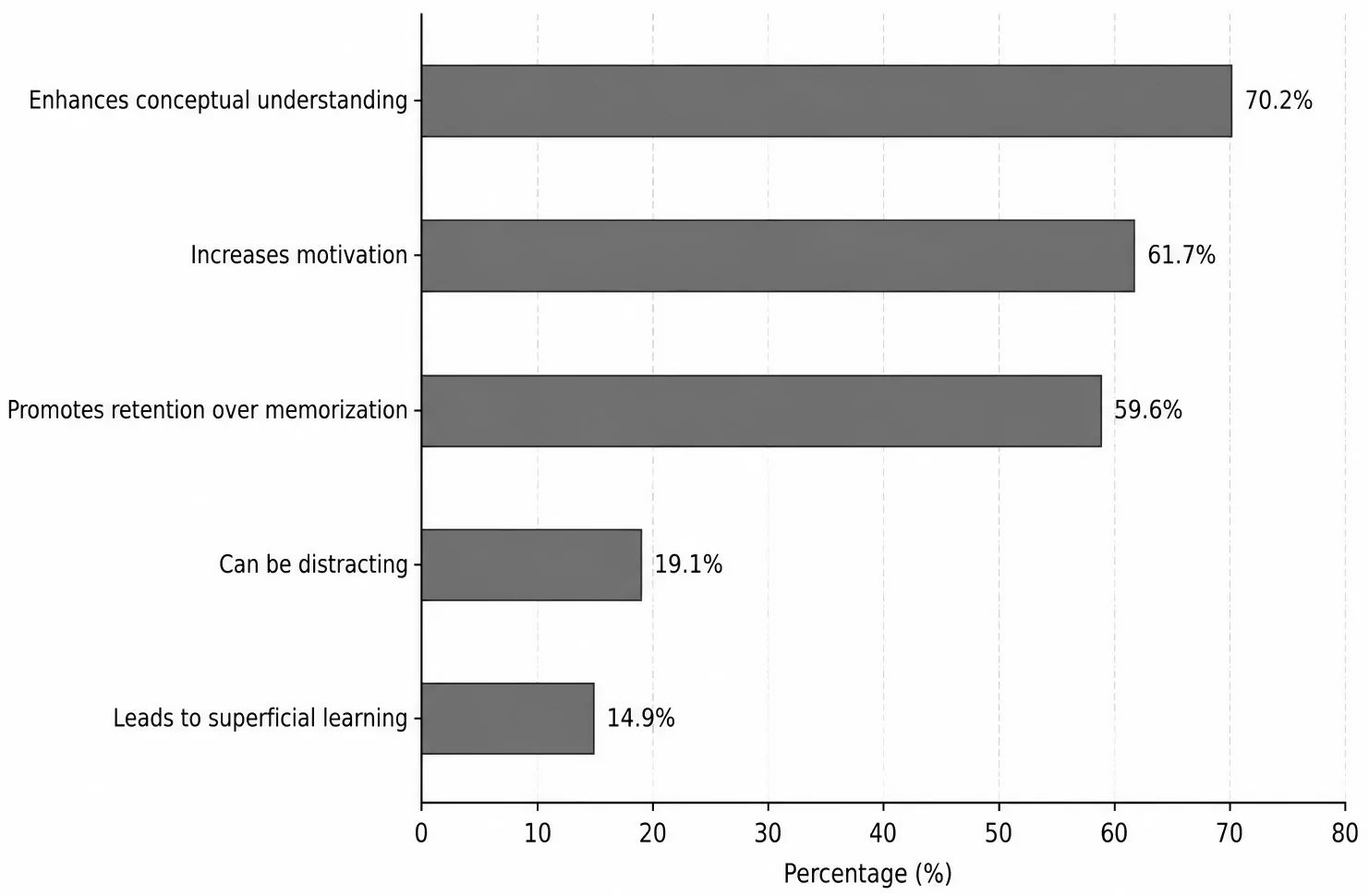
Student-Reported Perceptions of Digital Tools in Relation to Learning.

Alt text for Fig 3: Bar chart showing students’ self-reported perceptions of digital tools in relation to conceptual understanding, motivation, retention, distraction, and superficial learning.

For the questionnaire items presented in Fig 3, student responses were distributed as follows:

- 70.2% reported improved conceptual understanding.
- 61.7% reported increased learning motivation.
- 59.6% perceived that digital tools supported retention and meaning-based learning.
- 19.1% reported distraction during digital learning.
- 14.9% perceived occasional superficial engagement.

Representative student responses include:

“Animated explanations helped me finally hear sound relationships.” (Student 21)

“Switching between video platforms is easy, but sometimes I noticed that I couldn’t focus.” (Student 35)

### Overlaps and differences between student and instructor perspectives

To compare perspectives across groups, the categories derived from students’ and instructors’ open-ended responses were examined for similarities and differences, as described in the qualitative analysis procedure. The resulting cross-group comparison is summarized in Table 2.

**Table 2:** Comparative categories in student and instructor perceptions.

Comparative Category	Student Perspective	Instructor Perspective
Time and Pacing	Appreciates the flexibility to learn at own pace	Concerned that uncontrolled pacing may lead to superficial learning
Content Structure	Sees easy access and variety as beneficial	Questions the pedagogical consistency and depth of available content
Motivation	Finds digital platforms engaging and motivating	Sees motivational benefits but worries about potential loss of discipline
Depth of Learning	Values repeated, meaning-focused exposure	Observes tendencies toward shallow information acquisition
Purpose of Tool Use	Primarily uses tools for result-oriented problem solving	Prefers process-oriented, concept-building usage

The cross-group comparison showed both areas of convergence and divergence. Students primarily emphasized flexibility, repeated practice, accessibility, and motivation, whereas instructors more frequently emphasized pedagogical structure, depth of learning, and concerns about superficial or result-oriented use. Both groups identified potential benefits of digital and AI-based tools, while differing in the aspects of use they emphasized.

Representative participant responses include:

“Sometimes I expect deep understanding, but students are satisfied with the right answer.” (Teacher 2)

“When I reviewed it digitally at my own pace, I understood better.” (Student 38)

## Discussion

This study examined student and instructor perceptions of digital and artificial intelligence (AI)-supported tools in university-level music theory education. When the data obtained from the study are considered, both groups generally perceived potential benefits in the use of these technologies, although they differed in terms of the dimensions they emphasized. Students primarily emphasized flexibility, accessibility, repeated study, motivation, and the support provided for understanding musical concepts, whereas instructors placed greater emphasis on pedagogical structure, the value of learning, and the necessity of maintaining purposeful guidance during technology-supported learning. This distinction is quite important because it suggests that the educational value attributed to digital and AI-supported tools depends not only on the capacities of these tools, but also on how different users make sense of the role of these tools in the learning process, that is, on their individual meanings. This interpretation is consistent with the literature emphasizing that the adaptation or integration of technology in music and music theory education should focus not only on the availability of technological resources but also on pedagogical and educational purposes [19,34]. Current scientific studies and review studies similarly show that AI applications may provide new opportunities in terms of individualized or personalized instruction, feedback, practice, and interactive learning, while at the same time bringing pedagogical problems that require careful instructional evaluation [33,42].

Students’ responses to the questionnaire items were positive, particularly in terms of the perceived contributions of digital tools to conceptual understanding, motivation, and maintaining interaction with learning materials. In this study, 70.2% of the students reported that their conceptual understanding improved, 61.7% reported that their willingness to learn increased, and 59.6% reported that digital tools positively affected retention of information and meaning-oriented learning. These percentages should be interpreted as students’ perceptions rather than as improvements in objectively measured learning outcomes. Together with these findings, the emerging pattern also provides meaningful data in relation to previous research on technology-supported music teaching. For example, Uludag and Satir [18] found that students who participated in technology-supported music lessons had higher achievement in basic music theory than students who received traditional instruction; in their qualitative findings, they also reported increased motivation and willingness to study outside the classroom. Similarly, Wang and Liu [43] reported positive relationships among experiences, flow, and perceived learning efficiency in AI-based music education and emphasized the importance of emotional dimensions in terms of how individuals or students experience technology-supported music learning. Although the findings of the present study do not demonstrate similar causal effects, they show that students were able to associate digital learning environments with higher motivation and support for understanding issues related to music theory and theory.

In particular, the value students placed on being able to repeatedly access topics and content while studying may also be evaluated within established research on musical practice and self-discipline. Effective musical practice does not consist only of repetition; it may also include processes such as identifying places where mistakes are made, choosing appropriate personal strategies, monitoring performance, and evaluating progress. For example, Leon-Guerrero [7] identified repetition as the method most frequently used by adolescent instrumental students, but considered these practice behaviors within broader self-regulation processes. Hallam [1] and Hallam et al. [4] similarly determined that practice strategies and methods vary according to individual needs and developing levels of expertise. From this perspective, students’ preferences for reviewing materials repeatedly and working at their own pace in the present study may represent an important element of digital learning environments; however, repeated access to materials should not in itself be equated with deeper learning. Instead, the educational value of repetition may also depend on whether students use these opportunities strategically. For example, it is important that they use them to identify problems and difficulties, compare musical structures, correct errors, or monitor their own understanding. This distinction helps explain why, while students in the present study valued flexibility and repetition, instructors were also concerned about superficial or merely goal-oriented use.

Indeed, the findings also revealed a less positive aspect of students’ experiences. Although most of the responses emphasized perceived benefits, 19.1% of the participating students reported experiencing distraction during digital learning, while 14.9% reported occasionally perceiving superficial engagement. These findings make it difficult to interpret technology-supported learning only in positive terms. Easy access, rapid feedback, and student autonomy may facilitate participation; however, these do not necessarily guarantee sustained attention and conceptual depth. The qualitative responses also support this situation; while students supported the opportunity to review materials independently, they also stated that they had difficulty maintaining focus when moving between digital platforms. These findings are consistent with constructivist approaches to music technology that emphasize the importance of learning environments in which student autonomy is supported by appropriate educational structures. For example, Pierard and Lines [44] state that learning with music technology may extend from structured classroom activities to informal and self-directed practices and that teaching strategies responding to these different forms of participation are needed for digitally competent students. In this context, the current findings obtained from the study suggest that flexibility and autonomy are not inherently beneficial or harmful, but that their educational value is partly dependent on how independent exploration processes are structured and how the learning environment is related to learning purposes.

The instructor findings further support this interpretation. Although instructors generally acknowledged the potential of digital and AI-supported tools, they approached pedagogical consistency, depth of learning, and the tendency of students to prioritize reaching the correct answer over understanding the process leading to that answer more cautiously. The cross-group comparison therefore revealed an important difference in orientation: while students tended to evaluate the tools more in terms of ease of use, accessibility, motivation, and immediate benefit, instructors tended to evaluate them in terms of instructional sequencing, conceptual depth, and pedagogical control. This distinction does not necessarily mean that there is disagreement regarding the value attributed to technology. Rather, it reflects the different positions of the two groups within the learning environment. Bauer’s [34] framework of technology-supported music learning is particularly meaningful here because it conceptualizes effective technology integration through the relationship among technological, pedagogical, and content knowledge. In other words, access to technological functions alone does not constitute effective integration. Therefore, the emphasis placed by instructors in the present study on pedagogical structure may be interpreted as a concern regarding how technological opportunities are aligned with content knowledge and instructional goals.

The findings obtained from instructors should also be considered in terms of teachers’ readiness to integrate technology into the teaching process. Zhang, King, and Prior [21], in their study conducted with 307 music teachers, determined that technology acceptance directly influenced technological behavior, while being technologically competent had an indirect effect through technology acceptance. Although technology acceptance and competence were not determined through these constructs in the present study, teachers’ differing evaluations of digital and AI-supported resources and tools suggest that pedagogical integration may partly be related to how instructors evaluate the usefulness and appropriateness of specific technologies. This situation is also consistent with the broader literature suggesting that digital technologies should be considered not as educationally and pedagogically neutral transmission tools, but as tools that can support different forms of musical learning and participation. For example, the study conducted by Dobson and Littleton with undergraduate student musicians shows that digital technologies can mediate collaborative composition activities and can shape individuals’ creative practices. For this reason, the main question is not only whether technology is used, but also which musical and educational activities its use makes possible and how these activities are related to the intended learning processes.

The findings related to visual and interactive features provide another example of the relationship between technological possibilities and pedagogical goals. Participants associated these features, particularly when working with concepts that are not easy to convey only through verbal explanations, with musical structures becoming more accessible and understandable. These perceptions are generally consistent with review studies showing that digital technology is present and used at different levels and in different contexts of music education and can support various learning activities [8]. More recent AI-focused reviews extend this picture by identifying applications related to individualized learning, intelligent tutoring systems, interactive ear training, music theory, assessment, analysis, and other forms of technology-supported musical awareness and participation [19]. Nevertheless, the present findings indicate that technological possibilities should not be interpreted independently of instructional design. Visual and interactive presentations may facilitate access to materials; however, whether this turns into lasting and conceptual learning cannot be determined from self-report data. This situation requires longitudinal research and process-oriented studies.

Participants also expressed concerns regarding accessibility, language, cultural appropriateness, and the suitability of existing digital content. These concerns move the discussion beyond efficiency and ease of use. Although digital resources may be widely accessible through internet connectivity from a technological perspective, they may not be equally accessible pedagogically and culturally for particular groups of students. This issue is consistent with culturally responsive approaches to music education that demonstrate the relationship between learning experiences and individuals’ cultural and social contexts [45,46]. This issue is also becoming increasingly important in AI-supported music education. For example, Mazlan et al. [42] emphasize dataset bias, limited cultural sensitivity, and limitations in expressive feedback among the problems related to the use of AI applications in music education. These concerns are important for music education because musical knowledge and practices are embedded in cultural contexts. Therefore, the usefulness of an AI- and digitally supported resource should be evaluated not only through its technical performance, but also by considering which musical practices, terminologies, examples, and assumptions are represented within the system.

When the findings are evaluated as a whole, it can be seen that the main issue is not whether digital and AI-supported tools should be accepted or rejected in music and music theory education, but under which educational and pedagogical conditions they may be useful. While students’ emphasis on individuality, repeated access, accessibility, and motivation demonstrates meaningful advantages of these platforms, instructors’ concerns regarding superficial engagement, pedagogical consistency, rapid forgetting due to quick access to information, and conceptual depth point to the conditions under which these advantages may develop into meaningful learning. This interpretation is also consistent with current literature suggesting that digital and AI-supported music education may be more productive when technological opportunities are combined with human instruction than when they are used as an approach replacing human instruction. Mazlan et al. [42] describe hybrid approaches combining AI-supported analysis with human instruction as a promising direction for pedagogical practices. Within the scope of the present study, this situation suggests that digital and AI-supported tools may have a supportive and complementary role. While these tools provide new opportunities for practice, repetition, feedback, and exploration, instructors continue to bear responsibility for structuring learning goals, placing information in context, rapidly processing data, addressing misunderstandings, and supporting the establishment of theoretical and conceptual connections.

Several limitations should also be considered when interpreting these findings. First, the study was conducted in a limited context with 47 students and 9 instructors. Therefore, the findings should not be generalized to all university-level music education. In interpreting differences between groups, it should particularly be taken into account that the instructor sample constituted a smaller group. Second, the study primarily examined participants’ self-reported perceptions and experiences. Therefore, statements regarding conceptual understanding, motivation, remembering and retaining information, use of tools, and classroom practices represent participants’ own experiences rather than independently observed behaviors and/or objectively measured learning outcomes. Accordingly, the reported percentages should not be interpreted as strong evidence that digital and AI-supported tools cause improvements in learning. On the other hand, participants may have differed in terms of previous technological experiences, familiarity with specific applications, musical backgrounds, and frequency of technology use, and these factors may have influenced their evaluations. Finally, digital and AI-supported technologies are updated every day and show important differences in terms of their functions. Therefore, the findings should be evaluated in the context of the particular types of tools and forms of use reported by participants rather than being applied in the same way to all digital or AI systems. Future research conducted with larger and more diverse samples, and 10–15 years after the early period of widespread use of digital intelligence, may provide more meaningful, broader, and important data.

In general terms, this study shows that student and instructor perspectives provide evaluations of technology-supported music theory education that are not interchangeable but complementary, and that the study may also provide a basis for comparison in terms of historical continuity. While students emphasized flexibility, accessibility, repeated study, and motivation, instructors emphasized pedagogical structure, conceptual depth, and guided use. Considering these two perspectives together suggests that the pedagogical value of digital and AI-supported tools lies not so much in the technologies themselves as in the relationship among the opportunities offered by these technologies, the learning strategies of students and individuals, and instructors’ pedagogical decisions. In this context, it appears more appropriate to position these tools as resources that support and strengthen instructor-guided music theory instruction rather than replace it.

## Conclusion

From the learners’ perspective, digital platforms offer adjustable timing, clear visuals, and greater control over their own progress. When it comes to challenging concepts—such as modes, chord progressions, and musical form—students reported that interactive features and opportunities for repetition supported their understanding. These findings suggest that digital tools may broaden opportunities for repeated practice and engagement with music theory concepts. Yet the findings also indicate that access to digital practice opportunities alone may not necessarily lead to deeper understanding without structured feedback and purposeful engagement.

Meanwhile, certain students pointed out that using these tools without guidance might result in shallow learning experiences. Such observations support the view that technology alone may not ensure meaningful comprehension unless it is framed by intentional teaching strategies. Educators also expressed concerns—not only about the accuracy of material and alignment with course goals but also about growing student dependence on AI as an easy way to obtain answers instead of a path toward thoughtful analysis. Recent research on AI-supported music education similarly identifies pedagogical challenges concerning the reliability of AI-generated content, cultural sensitivity, and the need to combine technological support with human instruction [42]. What stands out here is a difference in emphasis: while learners often emphasize quick access and fast results, instructors place greater weight on thoroughness and methodical thinking.

The value of digital and AI-supported tools often relates to teaching purposes, not just their features. In music education, the educational value of technology depends partly on how technological possibilities are connected with pedagogical and content knowledge [34]. Poorly planned instruction can leave digital environments as additions rather than integral parts of the learning process. Language, culture, and clear learning targets matter if meaningful change is the aim. Tools gain educational value when they are aligned with purposeful pedagogical design.

Still, digital and AI-supported technologies may be most useful when they support, rather than substitute for, instructor-guided teaching and are carefully aligned with subject-specific goals. The findings of the present study suggest that instructors may shift from delivering information toward shaping guided encounters that link sound, symbols, and thought. Their educational value depends less on the presence of technology itself than on clear aims, mindful planning, and ongoing evaluation of practice.

## Supporting information

S1 File Minimal de-identified dataset. (XLSX)

S2 File Student questionnaire. (PDF)

S3 File Instructor questionnaire. (PDF)
