# Supplementary material for: The role of digital and AI-based tools in music theory education
Source: PLoS One. 2026 Nov 6;21(11):e0352494. doi: 10.1371/journal.pone.0352494 (PMC13742880; doi:10.1371/journal.pone.0352494)
Supplement: Supplementary file 1 [file pone.0352494.s002.pdf]

Effectiveness of Digital and Artificial Intelligence-Supported Tools in Music Theory Learning

Student Questionnaire

Informed Consent

This study is being conducted to examine students' views regarding the use of digital and artificial intelligence-supported tools in music theory learning. Participation in the study is voluntary.

Your responses to this questionnaire will be used solely for scientific research purposes and will be handled in accordance with principles of confidentiality. No personally identifiable information will be reported in the research findings. You may decline to participate or withdraw from the study before completing the questionnaire.

By selecting one of the options below, please indicate whether you have read the information provided above and voluntarily agree to participate in the study.

1. I have read the information provided above. Do you voluntarily agree to participate in the study?

☐ I agree
☐ I do not agree

A. Participant Information

2. Age:

....

3. Gender:

☐ Female
☐ Male
☐ Prefer not to say
☐ Other: ....

4. How many years have you been studying music theory?

.... years

5. How often do you use digital and/or artificial intelligence-supported tools (e.g., Chrome Music Lab, MuseScore, Finale, EarMaster, and ChatGPT) when learning music theory?

☐ Never
☐ Rarely

☐ Sometimes
☐ Often
☐ Very often

B. Student Views on Digital and Artificial Intelligence-Supported Tools

Instructions

The following statements concern the use of digital and artificial intelligence-supported tools in music theory learning. Please read each statement and select the option that best reflects your own view.

Response options:

1 = Strongly Disagree
2 = Disagree
3 = Neither Agree nor Disagree
4 = Agree
5 = Strongly Agree

S1. Digital tools allow me to progress at my own learning pace when studying music theory topics.

1 ☐ 2 ☐ 3 ☐ 4 ☐ 5 ☐

S2. Digital platforms make music theory concepts easier for me to understand by visualizing them.

1 ☐ 2 ☐ 3 ☐ 4 ☐ 5 ☐

S3. I find some digital and artificial intelligence-supported tools overly technical or mechanical to use.

1 ☐ 2 ☐ 3 ☐ 4 ☐ 5 ☐

S4. The limited availability of Turkish-language content in digital and artificial intelligence-supported tools makes my learning process more difficult.

1 ☐ 2 ☐ 3 ☐ 4 ☐ 5 ☐

S5. I think that digital and artificial intelligence-supported tools help me understand music theory topics conceptually.

1☐ 2☐ 3☐ 4☐ 5☐

S6. I think that digital and artificial intelligence-supported tools increase my motivation to learn music theory.

1☐ 2☐ 3☐ 4☐ 5☐

S7. I think that digital tools help me remember and make sense of the music theory topics I have learned.

1☐ 2☐ 3☐ 4☐ 5☐

S8. I think that I become distracted when studying music theory in digital environments.

1☐ 2☐ 3☐ 4☐ 5☐

S9. I think that, when using digital and artificial intelligence-supported tools, I sometimes engage with music theory topics superficially.

1☐ 2☐ 3☐ 4☐ 5☐

C. Open-Ended Questions

Please answer the following questions by considering your experiences of using digital and artificial intelligence-supported tools while learning music theory. Where possible, provide examples from your own experiences.

S10. Contributions

What do you think about the contributions of digital and artificial intelligence-supported tools to your music theory learning process? Please explain by providing examples from your experiences.

....
....
....
....

S11. Difficulties and Limitations

What difficulties or limitations have you encountered while using digital and artificial intelligence-supported tools to learn music theory? Please explain by providing examples from your experiences.

....
....
....
....

S12. Ways of Use

Please explain how and for what purposes you use digital and artificial intelligence-supported tools when learning music theory. Where possible, provide examples of how you use these tools.

....
....
....
....
