# Supplementary material for: The role of digital and AI-based tools in music theory education
Source: PLoS One. 2026 Nov 6;21(11):e0352494. doi: 10.1371/journal.pone.0352494 (PMC13742880; doi:10.1371/journal.pone.0352494)
Supplement: Supplementary file 2 [file pone.0352494.s003.pdf]

S3 File. Instructor Questionnaire

Effectiveness of Digital and Artificial Intelligence-Supported Tools in Music Theory Teaching

Instructor Questionnaire

Informed Consent

This study is being conducted to examine instructors' views regarding the use of digital and artificial intelligence-supported tools in music theory teaching. Participation in the study is voluntary.

Your responses to this questionnaire will be used solely for scientific research purposes and will be handled in accordance with principles of confidentiality. No personally identifiable information will be reported in the research findings. You may decline to participate or withdraw from the study before completing the questionnaire.

1. I have read the information provided above. Do you voluntarily agree to participate in the study?

☐ I agree
☐ I do not agree

A. Participant Information

2. Age:

....

3. Gender:

☐ Male
☐ Female
☐ Prefer not to say
☐ Other: ....

4. Highest Degree Obtained:

☐ Bachelor's degree
☐ Master's degree
☐ Doctoral degree
☐ Other: ....

5. Professional Experience:

.... years

6. Institution in Which You Work:

☐ Middle school
☐ High school
☐ University
☐ Other: ....

Purposes of Use and Perceived Limitations of Digital and Artificial Intelligence-Supported Tools

Instructions

The following statements concern the use of digital and artificial intelligence-supported tools in music theory teaching. Please read each statement and select the option that best reflects your own practices and views.

Response options:

1 = Strongly Disagree
2 = Disagree
3 = Neither Agree nor Disagree
4 = Agree
5 = Strongly Agree

IE1. I use digital and artificial intelligence-supported tools (e.g., Chrome Music Lab, MuseScore, Finale, EarMaster, and ChatGPT) to support students' independent study and practice outside the classroom.

1 ☐ 2 ☐ 3 ☐ 4 ☐ 5 ☐

IE2. I use digital and artificial intelligence-supported tools to diversify examples of harmonic analysis.

1 ☐ 2 ☐ 3 ☐ 4 ☐ 5 ☐

IE3. I use digital and artificial intelligence-supported tools to visually represent and explain musical structures.

1 ☐ 2 ☐ 3 ☐ 4 ☐ 5 ☐

IE4. Inadequate technical infrastructure or limited pedagogical materials make it difficult for me to use digital and artificial intelligence-supported tools in music theory teaching.

1 ☐ 2 ☐ 3 ☐ 4 ☐ 5 ☐

IE5. I think that students' use of digital and artificial intelligence-supported tools to obtain ready-made answers may lead to superficial learning.

1 ☐ 2 ☐ 3 ☐ 4 ☐ 5 ☐

Open-Ended Questions

Please answer the following questions by considering your experiences and observations regarding digital and artificial intelligence-supported tools in music theory teaching. Where possible, provide examples from your own experiences.

IE6. Contributions

What do you think about the contributions of digital and artificial intelligence-supported tools to music theory teaching and students' learning processes? Please explain by providing examples from your experiences or observations.

....
....
....
....

IE7. Difficulties and Limitations

What difficulties and limitations have you encountered or observed when using digital and artificial intelligence-supported tools in music theory teaching? Please explain by providing examples from your experiences.

....
....
....
....

IE8. Students' Ways of Using the Tools and the Learning Process

What have you observed regarding how and for what purposes students use digital and artificial intelligence-supported tools when learning music theory? Please explain, with examples, how these patterns of use affect students' learning processes.

....
....
....
....
